# Investigating the Impact of Asp181 Point Mutations on Interactions between PTP1B and Phosphotyrosine Substrate

**DOI:** 10.1038/srep05095

**Published:** 2014-05-28

**Authors:** Mengyuan Liu, Lushan Wang, Xun Sun, Xian Zhao

**Affiliations:** 1 State Key Laboratory of Crystal Materials, Shandong University, Jinan 250100, China; 2 State Key Laboratory of Microbial Technology, Shandong University, Jinan 250100, China

## Abstract

Protein tyrosine phosphatase 1B (PTP1B) is a key negative regulator of insulin and leptin signaling, which suggests that it is an attractive therapeutic target in type II diabetes and obesity. The aim of this research is to explore residues which interact with phosphotyrosine substrate can be affected by D181 point mutations and lead to increased substrate binding. To achieve this goal, molecular dynamics simulations were performed on wild type (WT) and two mutated PTP1B/substrate complexes. The cross-correlation and principal component analyses show that point mutations can affect the motions of some residues in the active site of PTP1B. Moreover, the hydrogen bond and energy decomposition analyses indicate that apart from residue 181, point mutations have influence on the interactions of substrate with several residues in the active site of PTP1B.

Protein tyrosine phosphatase 1B (PTP1B) plays a major role in insulin and leptin receptor dephosphorylation, suggesting that it acts as a key negative regulator of insulin and leptin signaling pathway^1^,^2^,^3^,^4^,^5^,^6^. Researches show that PTP1B-knockout mice exhibit increased insulin sensitivity and are resistant to diet-induced obesity, while treatment with PTP1B antisense oligonucleotides results in the improvement of hyperglycemia in diabetes mice models^7^,^8^,^9^. Therefore, PTP1B is an attractive target to treat type II diabetes and obesity^10^,^11^. Due to this reason, the X-ray crystal structures of PTP1B have been intensively studied, the results suggest that the active site of PTP1B consists of four regions (Figure 1): P loop^12^, WPD loop^13^,^14^, secondary aryl-phosphate-binding site^15^,^16^ and other residues^17^.

Among these residues, D181 is conserved and locates on WPD loop^18^. Previous experimental researches^18^,^19^ have reported that D181A and D181E point mutations enhance the binding affinity between PTP1B and substrate. It is deduced that D181 point mutations can have influence on the interactions of substrate with residues in the above four regions and lead to enhanced binding affinity. However, due to the lack of X-ray crystal structures of D181 mutants, the experimental researches about this issue are limited. Under this condition, molecular dynamics simulation may be useful to achieve this goal.

Considering this, the aim of this work is to determine the residues which interact with substrate can be affected by D181 point mutations and lead to increased substrate binding. To achieve this goal, wild type PTP1B/substrate complex and two mutants (D181A and D181E) were constructed to carry out molecular dynamics simulations. It is hoped that clarifying this issue can lead to deeper understanding of more factors influencing substrate binding.

## Results

### Stability of the complexes

The root mean square deviation (RMSD) of backbone Cα atoms from the starting structures of production dynamics are calculated and plotted in Figure 2A. As illustrated in Figure 2A, all systems deviate to a quite similar extent from their starting structures after 20 ns, resulting in a backbone RMSD of approximately 0.10–0.15 nm in the molecular dynamics simulations. The above data indicates that all of the systems reach equilibrium in the last 30 ns. Moreover, RMSD values of four regions (P loop, WPD loop, secondary aryl-phosphate-binding site and other residues) in the active site of PTP1B are also calculated (Supplementary Figure S1). The results suggest that D181A can influence the backbone stability of P loop, WPD loop regions and other residues in the active site of PTP1B. D181E can only affect the backbone stability of P loop and WPD loop regions.

The RMSF reflects the mobility of a certain residue around its mean position, which is another tool for studying the dynamics stability of the system. Figure 2B shows that both of the two mutations have influence the RMSF of residues 160–185 and 205–220. But it should be noted that D181A can have effect on the RMSF of residues 25–50, while D181E only affects the RMSF of residues 25–32. These results suggest that D181A can have influence on the conformation of P loop, WPD loop regions and other residues in the active site of PTP1B. D181E only affects the conformation of P loop and WPD loop regions.

### Cross-correlation analysis

To further investigate the effect of point mutations on the extent of correlation motions, cross-correlation matrices of the Cα atom fluctuations in the last 30 ns of the production runs were calculated and plotted in Figure 3. Highly positive regions (red and yellow) are associated with strong correlated motions of specific residues, whereas negative regions (dark blue) are indicative of strong anticorrelation in the specific residue movements. The results show that there are very few highly correlated motions except for the diagonal square, which reflects the correlation of a residue with itself. The anticorrelated motions of the WT are stronger than two mutants (blue). In wild type PTP1B/substrate complex, obvious anticorrelated motions are found in residues 40–76 and 130–175. Moreover, strong anticorrelated motions between 130–175 and 200–220 are also found (Figure 3A). Comparing with WT, D181A and D181E point mutations decrease the extent of anticorrelated motions in these residues (Figures 3B–C). This result suggests that the conformation nearby residues 40–76, 130–175 and 200–220 can be affected by point mutations.

### Principal component analysis

In order to investigate the significant motions in WT, D181A and D181E complexes, principal component analysis (PCA) are carried out. Figure 4A shows a plot of the eigenvalues obtained from the diagonalization of the covariance matrix of the atomic fluctuations. The first few eigenvalues are relative to concerted motions, and quickly decreased in amplitude to reach a number of constrained, more localized fluctuations. This analysis suggests that the first 20 principal components (PC) can account for 58.8%, 56.0% and 60.2% of the motions observed in the last 30 ns of the trajectories for WT, D181A and D181E, respectively. It can be seen from Figure 4A that the properties of the motions described by the first few PCs are different for the three systems. The magnitude of PC1 is decreased by both of the two point mutations.

In order to find the reasons how point mutations affect the motions described by PC1, the displacements of PC1 for the three complexes are calculated. Figure 4B suggests that D181A can influence the motions of residues 25–45, 160–185 and 200–220, while D181E can affect the motions of residues 25–30, 160–185 and 200–220. This indicates that D181A can have influence on the motions of P loop, WPD loop regions and other residues in the active site of PTP1B. D181E only affects the motions of P loop and WPD loop regions, which is consistent with the RMSF analysis.

### MM-PBSA calculation

In order to get deeper understanding of the effects of point mutations on the interactions between PTP1B and substrate, the binding free energies and the individual energy components are calculated by MM-PBSA method. Table 1 shows that D181A and D181E point mutations can enhance the binding affinity between PTP1B and substrate, which is accord with the previous experimental results^18^,^19^. Comparing the individual components contributing to the binding free energy (Table 1), it can be concluded that the ΔE_ele_ and ΔG_pol_ dominate the change in the binding strength.

### Analysis of the hydrogen bonds between PTP1B and substrate

Considering that the electrostatic interactions dominate the change in binding free energy caused by point mutations, the hydrogen bonding interactions between substrate and residues in PTP1B are investigated. During molecular dynamics simulations, the substrate can only form hydrogen bonds with S216, A217, G218, I219, G220 and R221. The hydrogen bonds between substrate and the above residues are analyzed (Table 2 and Supplementary Table S1). In WT and D181A complexes, the backbone N-H of A217, I219, G220 and R221, as well as the NE-HE in the guanidine group of R221, can exhibit two hydrogen bonds with the substrate phosphate group. When one hydrogen bond disappears, another hydrogen bonding occurs (Supplementary Table S1). Based on this, it can be inferred that the hydrogen bond probabilities of substrate with I219, G220 and R221 are all higher than 90% in WT and D181A complex. The hydrogen bond probability between substrate and A217 is about 80% for WT, while higher than 90% for D181A. Therefore, it can be concluded that D181A can increase the hydrogen bond stability of substrate with A217 and G218, while D181E leads to enhanced hydrogen bond stability between substrate and S216. These results suggest that D181A can enhance the hydrogen bond interactions of substrate with A217 and G218; D181E can lead to increased hydrogen bond interactions between substrate and S216. However, it must be noted that the change in hydrogen bonding probability is not enough to determine the variation of interactions between substrate and residues in PTP1B because this analysis only reflects the electrostatic interactions between substrate and residues. To more fully explore the change in interactions of substrate with residues in the PTP1B active site, the interaction energies between substrate and residues in PTP1B must be calculated.

### Substrate-residue interaction energy analysis

The total, electrostatic and van der Waals interaction energies between substrate and residues in PTP1B are calculated. The total interaction energies between substrate and residues are listed in Figure 5. The calculated results show that point mutations have significant effect on the interactions of substrate with residues in the active site of PTP1B.

In P loop region, D181A can result in increased interactions between substrate and G218. Though the hydrogen bond analysis suggests that D181A can make the hydrogen bond between substrate and A217 more stable than WT, the difference in the interaction energies between substrate and A217 caused by D181A is not significant. Unlike D181A, D181E leads to increased interactions between substrate and S216 (Figure 5).

In WPD loop region, D181A can enhance the interactions of substrate with F182. D181E results in increased interactions between substrate and residue 181 (Figure 5).

From the data in Figure 5, it can be seen that the differences of the interaction energies between substrate and residues in secondary aryl-phosphate-binding site are not significant.

Besides the above three regions, significant change in the interactions of substrate with other residues in the binding site are also found (Figure 5). D181A could enhance the interactions of substrate with Y46 and K120, while D181E has little effect on the interactions between substrate and these residues.

## Discussion

Figure 6A shows that the positively charged nitrogen atom of the substrate is close to the side chain of D181 in WT. Comparing with WT, the positively charged nitrogen atom of the substrate moves away from the side chain of A181 in D181A mutant (Figure 6B). This significant change then makes the phosphate group of substrate move close to A217 and G218. That may be the reasons for the increased hydrogen bond stability of substrate with A217 and G218. Unlike D181A, no significant change is found in the conformation of substrate (Figure 6C). But due to the increased side chain of E181, the electrostatic repulsion between residue 181 and the phosphate group of substrate becomes smaller. Then the phosphate group moves closer to S216 and forms more stable hydrogen bonds with this residue.

Though the hydrogen bond analysis suggests that D181A point mutation can enhance the stability of hydrogen bonds between substrate and A217, the difference in the interaction energies between substrate and A217 is not significant. A possible explanation is that the substrate moves too close to A217 in D181A mutant, which leads to increased van der Waals repulsions (Supplementary Figures S2–S3).

In WPD loop region, because of the conformation change of substrate in D181A mutant, the aromatic ring of substrate is much closer to F182 and result in enhanced hydrophobic interactions between substrate and F182 (Figure 6B). For D181E mutant, the positively charged nitrogen atom of the substrate moves closer to the carboxyl group of residue 181, which can increase the electrostatic attraction between substrate and E181 (Figure 6C).

For the other residues, D181A can lead to increased interactions of substrate with K120 because this mutation decreases the distance between the negatively charged oxygen atom of the substrate and positively charged nitrogen atom of K120. Compared with WT and D181E, it is noted that the positively charged nitrogen atom of substrate moves to the aromatic ring of Y46. This variation can result in favorable cation-π interactions, which enhances the interactions of substrate with Y46 (Figure 6B).

In conclusion, the cross-correlation and principal component analyses show that D181A can have influence on the motions of P loop, WPD loop regions and other residues in the active site of PTP1B. D181E only affects the motions of P loop and WPD loop regions. Hydrogen bond and energy decomposition analyses suggest that D181A can enhance the interactions of substrate with Y46, K120, F182 and G218. D181E can strengthen the interactions of substrate with residue 181 and S216.

## Methods

### Initial structure preparation

**Figure 1:**
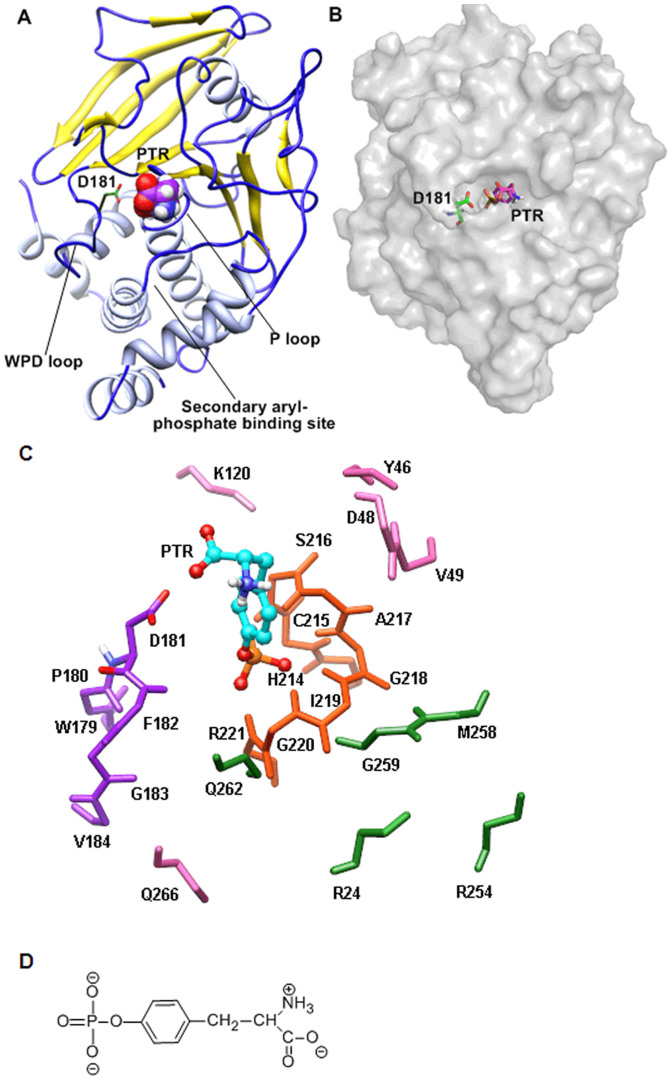
The structure of wild type PTP1B and substrate PTR. (A) Ribbon structure of PTP1B/substrate complex. (B) The protein surface of PTP1B/substrate complex. (C) The active site of wild type PTP1B. Residues in PTP1B are only shown with backbone atoms except D181. Substrate (PTR) is shown in ball and stick with carbon atoms in cyan. The P loop, WPD loop, secondary aryl-phosphate-binding site and other residues are shown in stick with atoms in yellow, purple, green and pink, respectively. Only polar hydrogen is displayed for clarity. (D) The chemical structure of substrate PTR used in molecular dynamics simulation.

**Figure 2:**
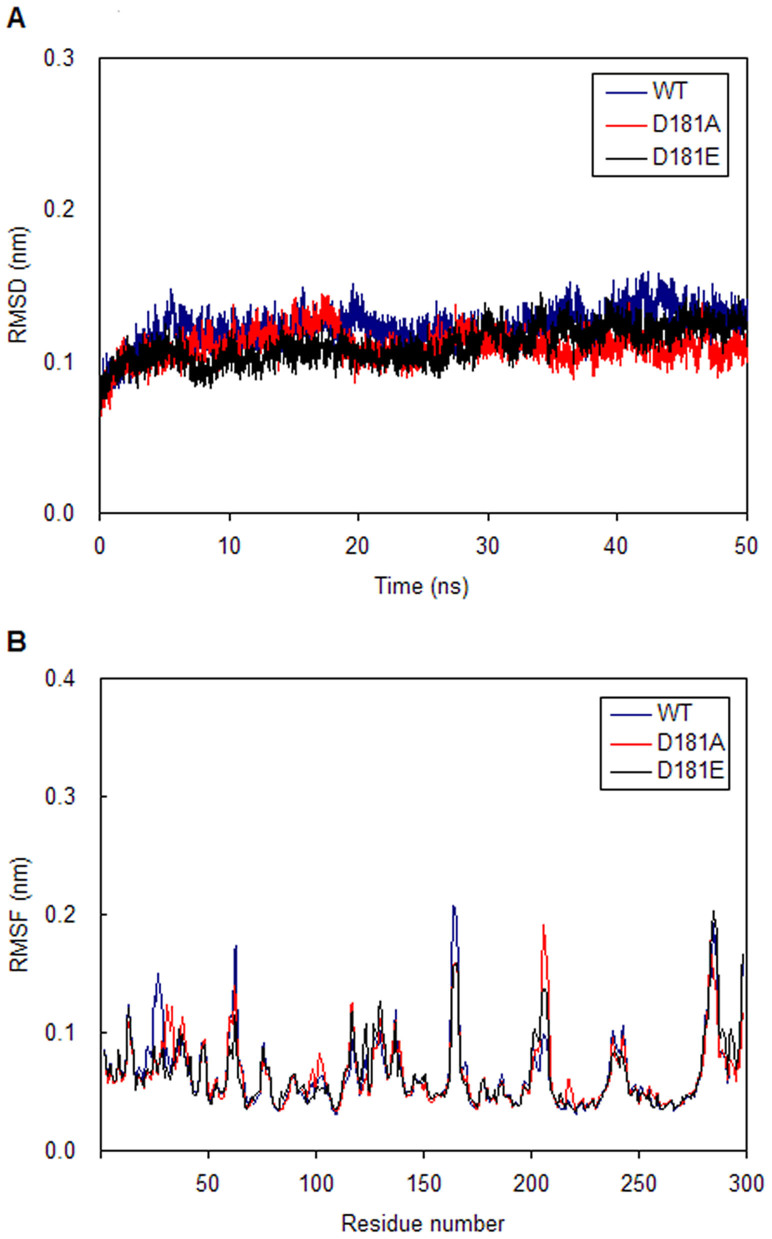
RMSD and RMSF values for wild type PTP1B and its mutants. (A) Time dependences of root mean square deviation (RMSD) of backbone Cα atoms from the initial structures of wild type PTP1B and its mutants. (B) The root mean square fluctuation (RMSF) for Cα atoms of wild type PTP1B and its mutants.

**Figure 3:**
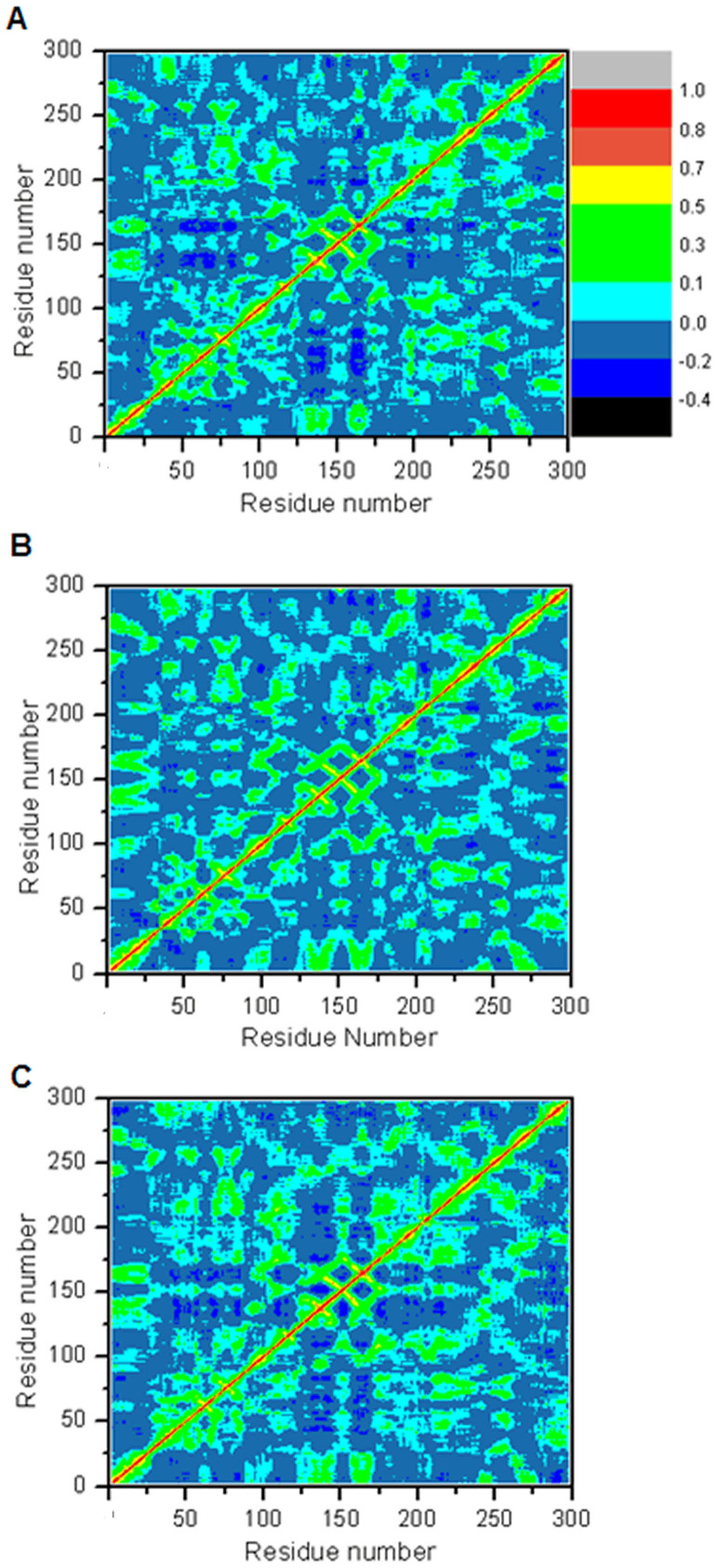
Cross-correlation matrices of the fluctuations of coordinates for Cα atoms around their mean positions during the last 30 ns of MD simulation. The extent of correlated motions and anticorrelated motions are colour-coded. (A) WT. (B) D181A. (C) D181E.

**Figure 4:**
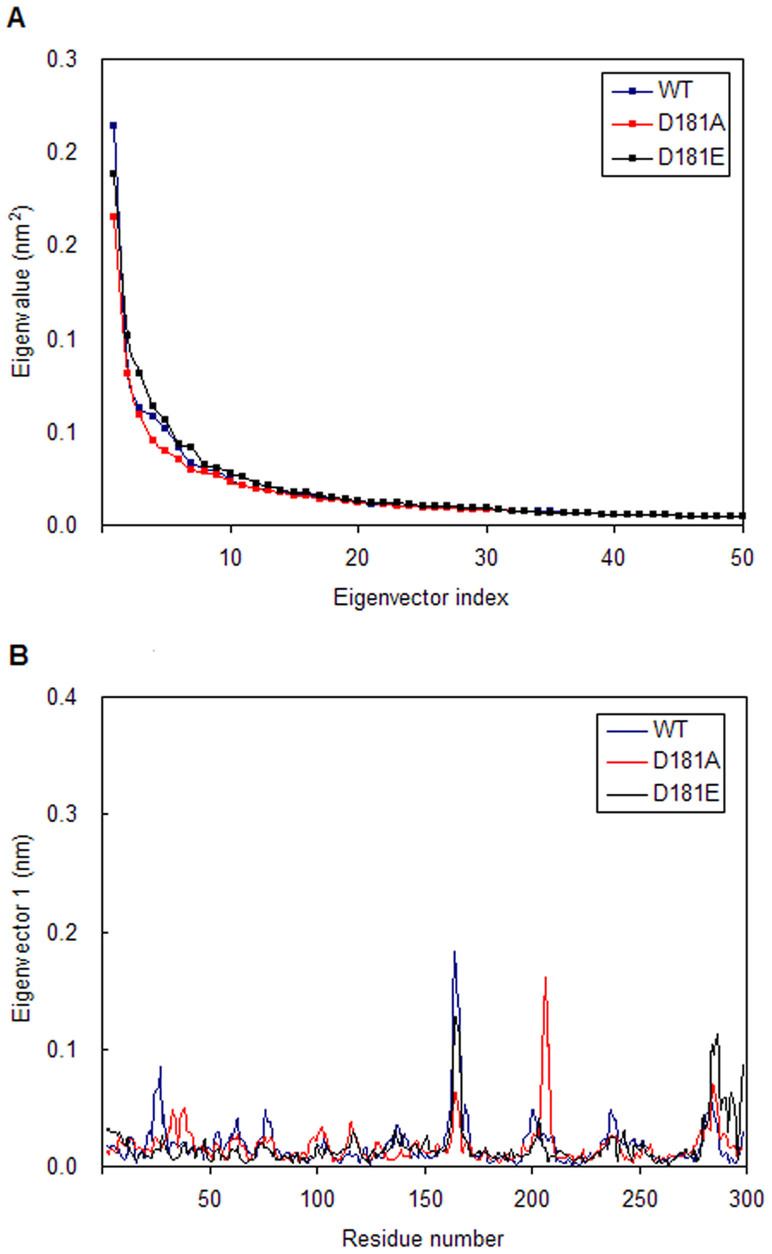
The results of principal component analysis. (A) The eigenvalues plotted against the corresponding eigenvector indices obtained from the Cα covariance matrix constructed from the last 30 ns of MD simulations. (B) Displacements of the components of the wild type and its mutants for the first eigenvector.

**Figure 5:**
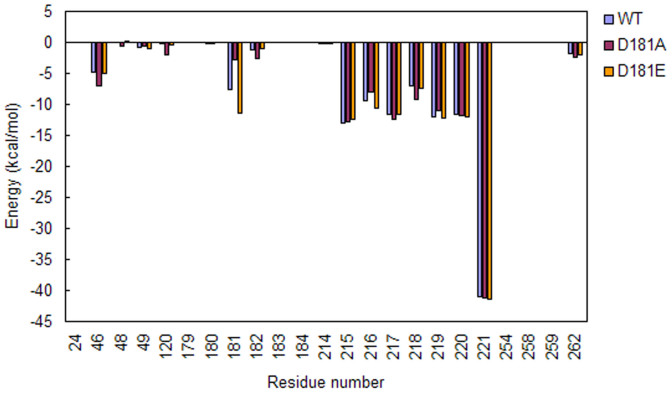
The average of total interaction energies between substrate and residues in the active site of PTP1B.

**Figure 6:**
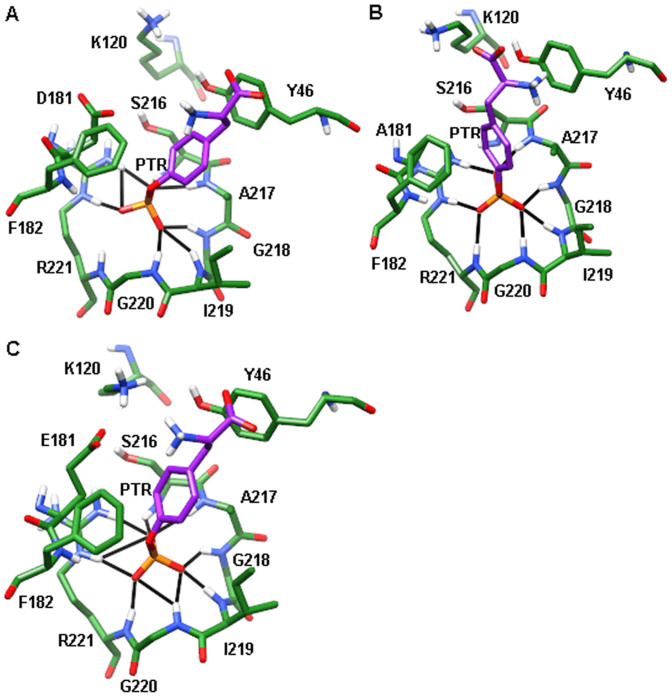
Snapshots of wild type PTP1B and its mutants after 50 ns molecular dynamics simulations. (A) WT. (B) D181A. (C) D181E. The hydrogen bonds are indicated by black line. Only polar hydrogen is displayed for clarity.

The structure of PTP1B complexed with substrate PTR (Figure 1D) was obtained from the Brookhaven Protein Data Bank (PDB code: 1PTV)^20^. Crystal water molecules within 4 Å of substrate were kept. A215 was then replaced by cysteine to get wild type PTP1B because the crystal structure was a C215A mutant. All of the hydrogen atoms were added by Maestro (Schrodinger LLC, New York). D181A and D181E mutants were also generated using Maestro software.

### Molecular dynamics simulations

Molecular dynamics simulations of the wild type PTP1B and its mutants were carried out using Gromacs 4.5.3 software^21^,^22^,^23^. The force field for proteins was Amber FF99SB^24^. The electrostatic potential of substrate PTR were calculated at HF/6-31G* level using NWChem 6.0^25^. The restrained electrostatic potential (RESP) method^26^ was then used for charge fitting. The remaining force field parameters for substrate PTR were taken from the general amber force field (GAFF)^27^. The complexes were then immersed in rectangular boxes containing TIP3P^28^ water molecules. And the box size was 8.367 × 7.201 × 6.124 nm. The sodium ions were added for charge neutralization. Particle Mesh Ewald (PME) method^29^,^30^ was used to treat long-range electrostatic interactions. To remove the steric clash, steepest descent energy minimization was first performed for the systems to give the maximum force below 1000 kJ·mol^−1^·nm^−2^. After that, the complexes were then equilibrated by 100 ps position restraint MD simulations with 1000 kJ·mol^−1^·nm^−2^ constant force on the heavy atoms of protein and substrate under NVT condition. 1 ns MD simulations without any restraint were sequentially carried out under NVT condition. Finally, 50 ns production molecular dynamics simulations were carried out under NVT condition. The temperature was kept at 300 K with V-rescale temperature coupling during the simulation process^31^. The LINCS algorithm^32^,^33^ was applied to constrain all bond lengths involving hydrogen atoms. Periodic boundary conditions were also employed during the molecular dynamics simulation. The time step was 1.0 fs. And the cut-off distance for van der Waals interaction was set to be 1.0 nm. The trajectories were sampled every 10 ps for analysis in production dynamics.

### Cross-correlation analysis

To investigate the extent of correlation motions caused by D181 point mutations, the cross-correlation matrix *C_ij_*, which reflected the fluctuations in the coordinates of the Cα atoms relative to their average positions from the last 30 ns of the simulations, was determined by the following equation^34^: 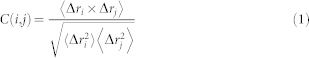
where the angle bracket represented an average over the sampled period and *Δr_i_* indicated the deviation of the Cα atom of the *i* th residue from its mean position. The value of *C_ij_* fluctuated from −1 to 1. Positive *C_ij_* values represented a correlated motion between the *i* th residue and the *j* th residue, while negative *C_ij_* values described an anticorrelated motion.

### Principal components analysis

The collective motions of wild type and mutated PTP1B were also investigated by principal components analysis (PCA)^35^. The positional covariance matrix *C* of atomic coordinates and its eigenvectors were used. The elements of the positional covariance matrix *C* were calculated by the following equation: 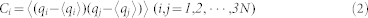
in which *q_i_* was the cartesian coordinate of the *i* th Cα atom, and *N* was the number of Cα atom in PTP1B. The average was calculated over the equilibrated trajectory after superimposition on a reference structure to remove overall translations and rotations by using a least-square fit procedure. The matrix *C* was symmetric and could be diagonalized by an orthogonal coordinate transformation matrix *T*, which transformed it into a diagonal matrix *Λ* of eigenvalues *λ_i_*: 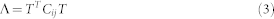
where the columns were the eigenvectors corresponding to the direction of motion relative to 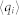, and each eigenvector associated with an eigenvalue that represented the total mean-square fluctuation of the system along the corresponding eigenvector. The last 30 ns production runs were used to perform this analysis.

### Hydrogen bond analysis

The hydrogen bond criteria used was an acceptor-donor distance of <0.35 nm, and acceptor…H-donor angle >120° 36. According to the literature^37^, the probability of hydrogen bond was calculated using following equation: 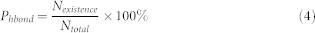
where *N_existence_* was the number of frames that targeted hydrogen bonds existed. *N_total_* was the total number of collected frames in production phase. The probability of each hydrogen bond was calculated in terms of a percentage that varied from 1% to 100%, where a percentage of 100 indicated that the hydrogen bond was highly stable and a percentage of 1 indicated an unstable hydrogen bond. 3000 snapshots isolated from the last 30 ns production runs with an interval of 10 ps were employed for hydrogen bond analysis. Besides, hydrogen bond information dumped for occupancies with dumping schematic of time series (every 60 ps) after each H-bond in the last 30 ns production runs was also calculated and listed in Supplementary Table S1.

### Binding free energy calculation

The molecular mechanics/Poisson-Boltzman surface area (MM/PBSA) method, which was implemented in Amber 12^38^, was applied to compute the binding free energies between PTP1B and substrate. In this method^39^, the binding free energies *ΔG_bind_* was calculated by the following equation: 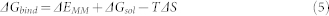
where *ΔE_MM_* was the molecular mechanics interaction energy, *ΔG_sol_* was the solvation free energy and −*TΔS* was the **entropy contribution**.

*ΔE_MM_* was the sum of electrostatic (*ΔE_ele_*) and van der Waals (*ΔE_vdw_*) interaction energies between PTP1B and substrate as follows: 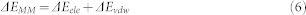
The solvation free energy contribution can be decomposed in two parts, the electrostatic (*ΔG_pol_*) and the nonpolar (*ΔG_nonpol_*) terms: 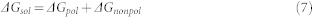
The interior and exterior dielectric coefficients were set to 1 and 80, respectively. The nonpolar contribution of the solvation free energy was computed as a function of the solvent accessible area (SAS), as follows: 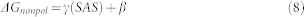
In this equation, *γ* = 0.00542 kcal/mol**·**Å^2^ and *β* = 0.92 kcal/mol. The SAS was estimated using a 1.4 Å solvent probe radius. The *ΔE_MM_* and *ΔG_sol_* calculations were performed using the same snapshots as the hydrogen bond analysis.

The normal-mode analysis was performed to compute the entropy contributions^40^. However, due to entropy calculations for large systems being extremely time-consuming, only 600 snapshots taken at an interval of 50 ps from the last 30 ns production runs were used to carry out this calculation. Each snapshot was minimized using a maximum of 10,000,000 steps and a root-mean-square (rms) gradient of 1 × 10^−4^ kcal/mol**·**Å.

### Substrate-residue interaction energy calculation

The electrostatic and van der Waals interaction energies between substrate and residues in PTP1B were calculated according to the Amber force field equation^41^. All energy components were calculated using the same snapshots as the hydrogen bond analysis.

## Author Contributions

M.L. wrote the manuscript and carried out molecular dynamics simulations. L.W., X.S. and X.Z. designed the experiments and revised manuscript.

## Supplementary Material

Supplementary Information Supplementary Data

## Figures and Tables

**Table 1:** Binding free energies and individual energy term between PTP1B and substrate calculated with MM-PBSA (Unit: kcal/mol)

Component	WT	D181A	D181E
ΔE_ele_^a^	−321.3	−384.8	−321.6
ΔE_vdw_^b^	−25.7	−24.3	−25.6
ΔG_pol_^c^	290.5	348.9	287.9
ΔG_nonpol_^d^	−3.6	−3.7	−3.7
−TΔS	21.4	22.0	21.5
ΔG_bind_^e^	−38.7	−41.9	−41.5

^a^Electrostatic interaction energies between PTP1B and substrate.

^b^van der Waals interaction energies between PTP1B and substrate.

^c^Polar contributions to the solvation free energy.

^d^Nonpolar contributions to the solvation free energy.

^e^ΔG_bind_ = ΔE_ele_ + ΔE_vdw_ + ΔG_pol_ + ΔG_nonpol_ − TΔS.

**Table 2:** The hydrogen bonds between substrate and key residues in PTP1B

Complex	Donor	Acceptor	Distance (Å)^a^	Angle (°)^a^	Probability (%)
WT	S216-N-H	PTR-O3P	2.91	144.25	56.51
	A217-N-H	PTR-O1P	3.10	161.41	20.26
	A217-N-H	PTR-O3P	3.21	160.14	63.85
	G218-N-H	PTR-O3P	2.99	131.81	16.76
	I219-N-H	PTR-O2P	2.98	163.46	80.31
	I219-N-H	PTR-O3P	2.90	163.47	14.26
	G220-N-H	PTR-O2P	2.99	161.38	79.07
	G220-N-H	PTR-O3P	2.96	159.26	14.26
	R221-N-H	PTR-O1P	2.95	166.53	80.91
	R221-N-H	PTR-O2P	2.99	165.10	14.16
	R221-NE-HE	PTR-O1P	3.00	161.24	84.51
	R221-NE-HE	PTR-O2P	2.99	158.83	15.53
	R221-NH2-HH21	PTR-O1P	3.00	153.64	21.69
	R221-NH2-HH21	PTR-O3P	2.80	163.60	82.07
D181A	S216-N-H	PTR-O3P	2.91	137.06	30.02
	A217-N-H	PTR-O2P	3.12	153.94	21.39
	A217-N-H	PTR-O3P	3.06	162.49	75.41
	G218-N-H	PTR-O2P	2.96	137.99	64.35
	I219-N-H	PTR-O1P	2.86	155.97	14.86
	I219-N-H	PTR-O2P	2.91	156.18	76.34
	G220-N-H	PTR-O1P	3.07	148.69	23.59
	G220-N-H	PTR-O2P	2.94	162.58	76.81
	R221-N-H	PTR-O1P	3.00	165.61	75.87
	R221-N-H	PTR-O3P	3.02	161.02	14.76
	R221-NE-HE	PTR-O1P	2.92	163.57	79.44
	R221-NE-HE	PTR-O2P	3.07	153.89	12.50
	R221-NH2-HH21	PTR-O1P	3.18	143.25	25.22
	R221-NH2-HH21	PTR-O3P	2.82	163.38	79.81
D181E	S216-N-H	PTR-O3P	2.89	144.44	87.70
	A217-N-H	PTR-O3P	3.18	161.85	89.14
	G218-N-H	PTR-O2P	3.08	131.93	18.66
	I219-N-H	PTR-O2P	2.91	163.59	99.90
	G220-N-H	PTR-O2P	2.92	163.22	99.30
	R221-N-H	PTR-O1P	2.98	166.80	99.53
	R221-NE-HE	PTR-O1P	2.91	165.39	99.47
	R221-NH2-HH21	PTR-O1P	3.38	131.69	23.56
	R221-NH2-HH21	PTR-O3P	2.80	163.55	99.93

^a^The hydrogen bonds are determined by the acceptor…donor atom distance of <0.35 nm and acceptor…H-donor angle of >120 Å.
