# Supplementary material for: Investigating the Impact of Asp181 Point Mutations on Interactions between PTP1B and Phosphotyrosine Substrate
Source: Sci Rep. 2014 May 28;4:5095. doi: 10.1038/srep05095 (PMC4035576; doi:10.1038/srep05095)
Supplement: Supplementary Information — Supplementary Data [file srep05095-s1.pdf]

Supplementary information

Investigating the Impact of Asp181 Point Mutations on Interactions between PTP1B and Phosphotyrosine Substrate

Mengyuan Liu [1], Lushan Wang [2], Xun Sun [1,] *, Xian Zhao [1,] *

[1] State Key Laboratory of Crystal Materials, Shandong University, Jinan 250100, China,

[2] State Key Laboratory of Microbial Technology, Shandong University, Jinan 250100, China.

Correspondence and requests for materials should be addressed to X. S. (sunxun@icm.sdu.edu.cn) and X. Z. (zhaoxian@icm.sdu.edu.cn)

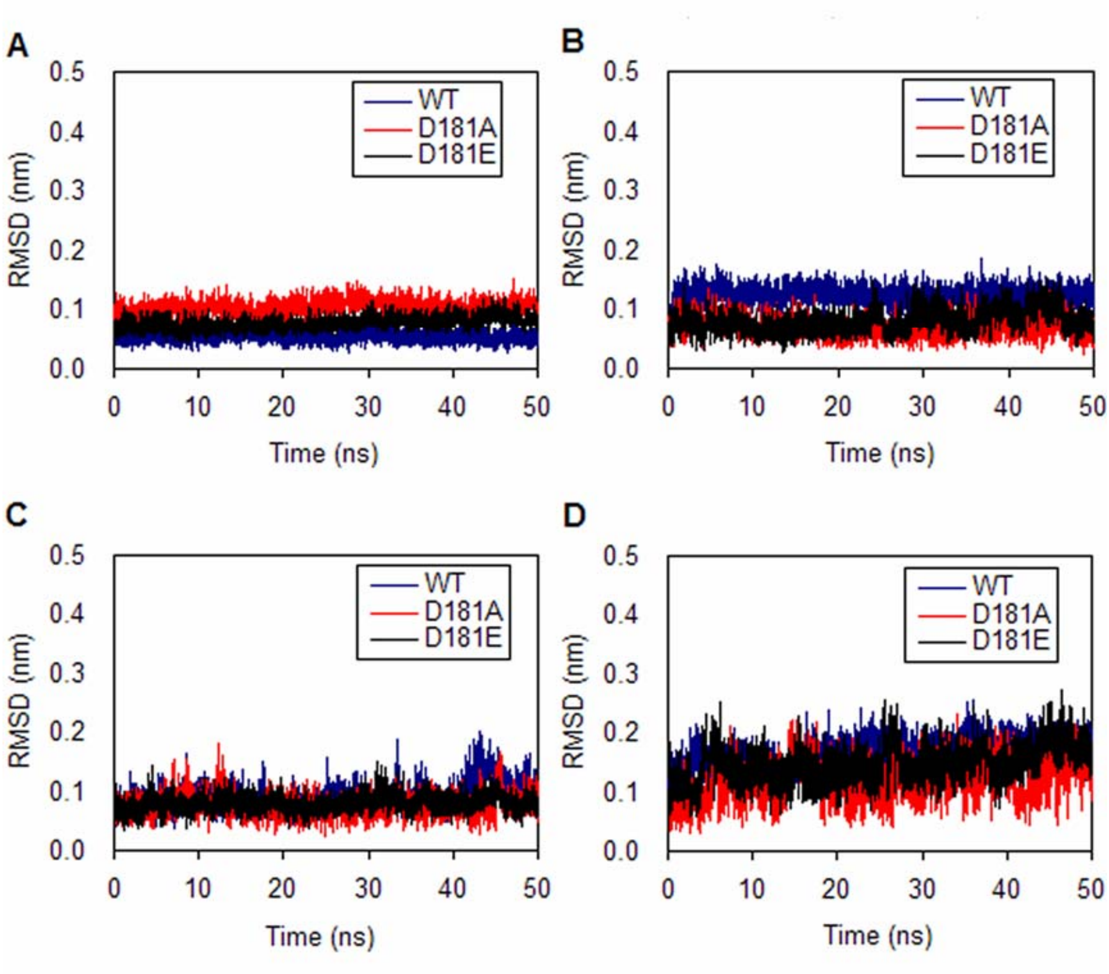

Figure S1 | Comparison of RMSD values in different regions. (A) P loop. (B) WPD loop. (C) Secondary aryl-phosphate-binding site. (D) Other residues in the active site of PTP1B.

Table S1 The analysis of hydrogen bonds between PTP1B and substrate during the last 30 ns MD simulations.

<table>
<tr><th>Complex</th><th>Donor</th><th>Acceptor</th><th>Distribution [a]</th></tr>
<tr><td rowspan="14">WT</td><td>S216-N-H</td><td>PTR-O3P</td><td>|xxx   oxxx*xxoxxxxxxxxxxxxxx***xooxxxooxxxox|</td></tr>
<tr><td>A217-N-H</td><td>PTR-O1P</td><td>| ..*@@@@@*. |</td></tr>
<tr><td>A217-N-H</td><td>PTR-O3P</td><td>|*xx ..-.---x**xxxxxx**xx*xxx*xxxxxxxxxxx**xxx*xx@|</td></tr>
<tr><td>G218-N-H</td><td>PTR-O3P</td><td>| ooo-ooo |</td></tr>
<tr><td>I219-N-H</td><td>PTR-O2P</td><td>|@@x   *@@@@@@@@@@@@@@@@@@@@@@@@@@*@@@@@@@@@@|</td></tr>
<tr><td>I219-N-H</td><td>PTR-O3P</td><td>| *@@@@@@. |</td></tr>
<tr><td>G220-N-H</td><td>PTR-O2P</td><td>|@@x ..-.-..*@@**@@*@@@@@@@*@@@**@@***@*x@@@**@@@@|</td></tr>
<tr><td>G220-N-H</td><td>PTR-O3P</td><td>| ..*@@@@@@. |</td></tr>
<tr><td>R221-N-H</td><td>PTR-O1P</td><td>|@@x   *@@@@@@@@@@@@@@@@@@@@@@@@@@@@@@@@@@@@@|</td></tr>
<tr><td>R221-N-H</td><td>PTR-O2P</td><td>| *@@@@@@. |</td></tr>
<tr><td>R221-NE-HE</td><td>PTR-O1P</td><td>|@@x -oo-xoo*@@**@@*@@@@@@@@@@@**@@@@*@**@@@**@@@@|</td></tr>
<tr><td>R221-NE-HE</td><td>PTR-O2P</td><td>| .--@@@@@@@. |</td></tr>
<tr><td>R221-NH2-HH21</td><td>PTR-O1P</td><td>|... *@@@@@@.... . ...-|</td></tr>
<tr><td>R221-NH2-HH21</td><td>PTR-O3P</td><td>|@@*-.   *@@@@@@@@@@@@@@@@@@@@@@@@@@@@@@@@@@@@@|</td></tr>
<tr><td rowspan="14">D181A</td><td>S216-N-H</td><td>PTR-O3P</td><td>|x-o-xxooo.-ooooooo-.----o---.   ---.o--o-o|</td></tr>
<tr><td>A217-N-H</td><td>PTR-O2P</td><td>|. ... .-@@@@@@@   .... |</td></tr>
<tr><td>A217-N-H</td><td>PTR-O3P</td><td>|@@@@*@@**@@@*****@@@@@@@@@@@x   ....x@@@@@@@@@|</td></tr>
<tr><td>G218-N-H</td><td>PTR-O2P</td><td>|**************x*xx*@**@x**x*x   oxx***xx**|</td></tr>
<tr><td>I219-N-H</td><td>PTR-O1P</td><td>| -@@@@@@@ |</td></tr>
<tr><td>I219-N-H</td><td>PTR-O2P</td><td>|@@@@@@@@@@@@@@@@@@@@@@@@@@@@x   x@@@@@@@@@|</td></tr>
<tr><td>G220-N-H</td><td>PTR-O1P</td><td>|...-..... .....-.. ..-. ...o@@@@@@@   . . ..-..|</td></tr>
<tr><td>G220-N-H</td><td>PTR-O2P</td><td>|@@@@@@@@@@@@@@@*@@@@@@@@@@@@x   . ..x@@@@@@@@@|</td></tr>
<tr><td>R221-N-H</td><td>PTR-O1P</td><td>|@@@@@@@@@@@@@@@@@@@@@@@@@@@@x   x@@@*@@@@@|</td></tr>
<tr><td>R221-N-H</td><td>PTR-O3P</td><td>| -@*@@@@@ |</td></tr>
<tr><td>R221-NE-HE</td><td>PTR-O1P</td><td>|@@@@@@@@@@@@@@@@@@@@@@@@@@@@x   -o-o*@@@@@@@@@|</td></tr>
<tr><td>R221-NE-HE</td><td>PTR-O2P</td><td>| .--.-.--@@@@- |</td></tr>
<tr><td>R221-NH2-HH21</td><td>PTR-O1P</td><td>|.--..o----.........--.--.---.   @@@@oo---....-|</td></tr>
<tr><td>R221-NH2-HH21</td><td>PTR-O3P</td><td>|@@@@@@@@@@@@@@@@@@@@@@@@@@@@x---.--.   x@@@@@@@@@|</td></tr>
<tr><td rowspan="9">D181E</td><td>S216-N-H</td><td>PTR-O3P</td><td>|xxx*x***xx**x*****@@******x*@***@***@@******@@*@@*|</td></tr>
<tr><td>A217-N-H</td><td>PTR-O3P</td><td>|@@*@@@*@*@@@***@x@*x*******@*@*x*******x***x******|</td></tr>
<tr><td>G218-N-H</td><td>PTR-O2P</td><td>|......-..............-..--...----.-----..-..----.-|</td></tr>
<tr><td>I219-N-H</td><td>PTR-O2P</td><td>|@@|</td></tr>
<tr><td>G220-N-H</td><td>PTR-O2P</td><td>|@@@@@@@@@@@@@@@@@@@@@@@@@@@@@@@@@@@@@@*@@@@@@@@@@@|</td></tr>
<tr><td>R221-N-H</td><td>PTR-O1P</td><td>|@@|</td></tr>
<tr><td>R221-NE-HE</td><td>PTR-O1P</td><td>|@@|</td></tr>
<tr><td>R221-NH2-HH21</td><td>PTR-O1P</td><td>|---..-.-----.---.--.--.--..--.--.----. -..-------.|</td></tr>
<tr><td>R221-NH2-HH21</td><td>PTR-O3P</td><td>|@@|</td></tr>
</table>

[a] Hydrogen bond information dumped for occupancies with dumping schematic of time series (every 60 ps) after each H-bond, key follows: “ ” 0-5%; “.” 5-20%; “-” 20-40%; “o”: 40-60%; “x”: 60-80%; “*”: 80-95%; “@”: 95-100%

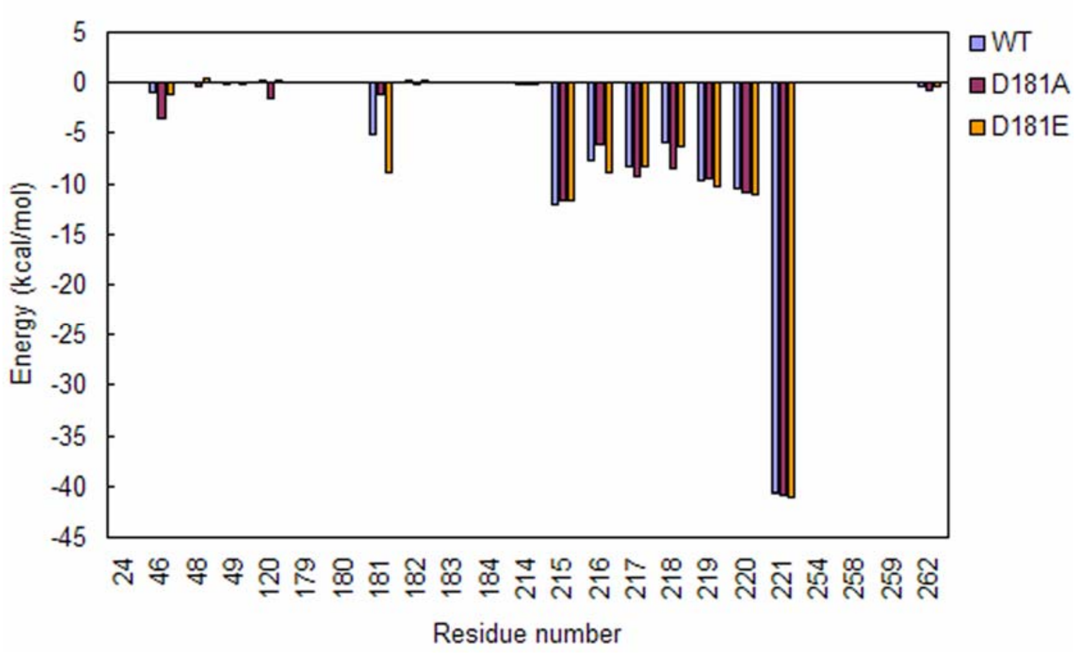

Figure S2 | The average of electrostatic interaction energies between substrate and residues in the active site of PTP1B.

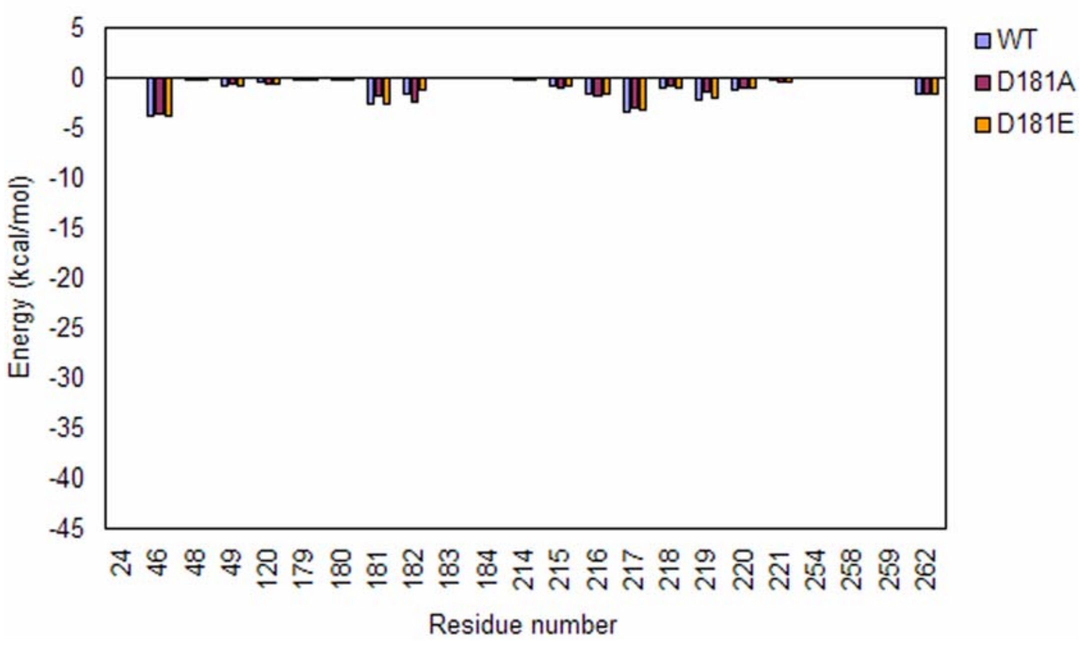

Figure S3 | The average of van der Waals interaction energies between substrate and residues in the active site of PTP1B.
